# Impact of Microparticle Transarterial Chemoembolization (mTACE) on myeloid‐derived suppressor cell subtypes in hepatocellular carcinoma: Clinical correlations and therapeutic implications

**DOI:** 10.1002/iid3.70007

**Published:** 2024-09-02

**Authors:** Yuanxun Yue, Zhizhong Ren, Yaqin Wang, Ying Liu, Xiaowei Yang, Tianxiao Wang, Yating Bai, He Zhou, Qian Chen, Sujun Li, Yuewei Zhang

**Affiliations:** ^1^ Department of Interventional and Pain, Beijing Luhe Hospital Capital Medical University Beijing China; ^2^ Hepatobiliary Interventional Department Beijing Tsinghua Chang Gung Hospital Affiliated to Tsinghua University Beijing China; ^3^ Thorgene Co., Ltd. Beijing China; ^4^ Shanghai Dengding BioAI Co. Shanghai China; ^5^ Translational Medicine Institute of Jiangxi, The First Affiliated Hospital of Nanchang University Nanchang China; ^6^ JiangXi Key Laboratory of Transfusion Medicine Nanchang China

**Keywords:** early stage MDSCs, gelatin sponge microparticles (GSMs), hepatocellular carcinoma (HCC), monocytic‐myeloid‐derived suppressor cells (mMDSCs), Transarterial Chemoembolization (TACE)

## Abstract

**Background:**

Myeloid‐derived suppressor cells (MDSCs) play a pivotal role in immunosuppression and tumor progression in hepatocellular carcinoma (HCC). While various treatments like surgical resection, ablation, and radiotherapy have been studied for their effects on circulating MDSC frequencies in HCC patients, the findings remain inconclusive. Transarterial Chemoembolization (TACE) stands as the standard care for unresectable HCC, with Microparticle TACE (mTACE) gaining prominence for its capacity to induce significant tumor necrosis. However, the immunological ramifications of such pathological outcomes are scarcely reported.

**Methods and Results:**

This study aims to elucidate the alterations in MDSC subtypes, specifically monocytic MDSCs (mMDSCs) and early‐stage MDSCs (eMDSCs), post‐mTACE and to investigate their clinical correlations in HCC patients. A cohort comprising 75 HCC patients, 16 liver cirrhosis patients, and 20 healthy controls (HC) was studied. Peripheral blood samples were collected and analyzed for MDSC subtypes. The study also explored the associations between MDSC frequencies and various clinical parameters in HCC patients. The frequency of mMDSCs was significantly elevated in the HCC group compared to liver cirrhosis and HC. Importantly, mMDSC levels were strongly correlated with aggressive clinical features of HCC, including tumor size, vascular invasion, and distant metastasis. Post‐mTACE, a marked reduction in mMDSC frequencies was observed, while eMDSC levels remained stable.

**Conclusions:**

Our findings underscore the critical role of mMDSCs in HCC pathogenesis and their potential as a therapeutic target. The study also highlights the efficacy of mTACE in modulating the immunosuppressive tumor microenvironment, thereby opening new avenues for combinatorial immunotherapeutic strategies in HCC management.

## Highlights

- The study underscores the pivotal role of myeloid‐derived suppressor cells (MDSCs) in driving immunosuppression and tumor progression in hepatocellular carcinoma (HCC).
- It highlights the inconclusive findings regarding the effects of various HCC treatments on MDSC frequencies, including surgical resection, ablation, and radiotherapy.
- The study delves into the immunological consequences of Microparticle Transarterial Chemoembolization (mTACE), a treatment gaining prominence for inducing significant tumor necrosis in unresectable HCC.
- It investigates the clinical correlations between MDSC frequencies, specifically monocytic MDSCs (mMDSCs), and aggressive HCC features like tumor size, vascular invasion, and distant metastasis.
- The research highlights the therapeutic potential of targeting mMDSCs and demonstrates the effectiveness of mTACE in reshaping the immunosuppressive tumor microenvironment. This offers new avenues for innovative immunotherapeutic approaches in HCC management.

## Graphical abstract

The research highlights the therapeutic potential of targeting monocytic myeloid‐derived suppressor cells (mMDSCs) and demonstrates the effectiveness of Microparticle TACE (mTACE) in reshaping the immunosuppressive tumor microenvironment. This offers new avenues for innovative immunotherapeutic approaches in hepatocellular carcinoma (HCC) management. It investigates the clinical correlations between MDSC frequencies, specifically mMDSCs, and aggressive HCC features like tumor size, vascular invasion, and distant metastasis. The study delves into the immunological consequences of mTACE, a treatment gaining prominence for inducing significant tumor necrosis in unresectable HCC.

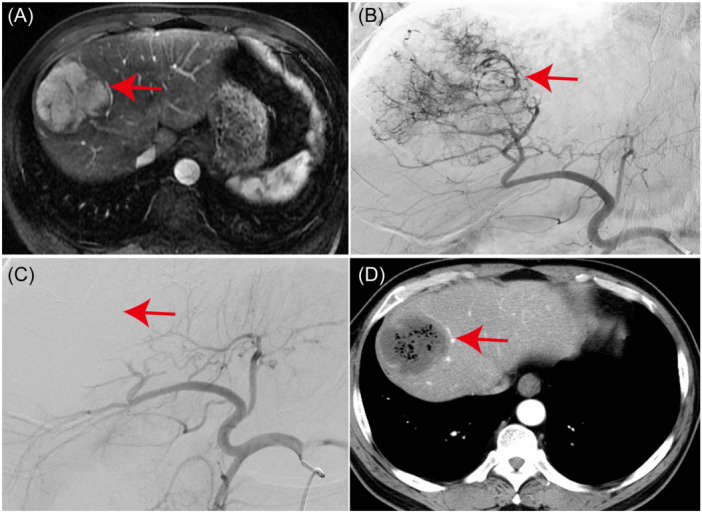

## 1 INTRODUCTION

Hepatocellular carcinoma (HCC) ranks as the sixth most prevalent cancer and stands as the third leading cause of cancer‐related mortality globally.^1^ Myeloid‐derived suppressor cells (MDSCs) are a heterogeneous population of immature myeloid cells with potent immunosuppressive capabilities, thereby facilitating tumor metastasis.^2^ Among the MDSC subtypes, monocytic MDSCs (mMDSCs) constitute a significant proportion in humans, while early‐stage MDSCs (eMDSCs) represent a minor but potent subset, accounting for less than 5% of the total MDSC population.^3^ Extensive research has demonstrated the detrimental impact of MDSCs on antitumor immunity, revealing that MDSCs depletion enhances effector T‐cell populations and restores T‐cell functionality.^4^, ^5^ Clinical studies further corroborate that a reduced frequency of MDSCs is associated with improved clinical outcomes in HCC patients.^6^, ^7^ Current therapeutic strategies for HCC encompass surgical resection, radiofrequency ablation (RFA), transcatheter arterial chemoembolization (TACE), and transarterial radioembolization (TARE) with Yttrium‐90, each tailored to the specific stage of the disease.^8^, ^9^ Microparticle‐TACE (mTACE) is distinguished by its use of particulate embolic agents exclusively for embolizing tumor‐feeding arteries. In our clinical practice, we have observed significant tumor necrosis, including liquefied necrosis, particularly in cases of large HCC tumors.^10^, ^11^ However, the implications of such tumor necrosis on the frequency of MDSC subtypes remain largely unexplored.

In light of this, the present study aims to investigate the alterations in mMDSCs and eMDSCs frequencies in HCC patients following mTACE treatment. Additionally, we compare the frequencies of these MDSC subtypes among HCC patients, individuals with liver cirrhosis, and healthy controls (HC). Finally, we analyze the associations between mMDSCs, eMDSCs, and various clinical parameters in HCC patients.

## 2 MATERIALS AND METHODS

### 2.1 Participants

Patients diagnosed with HCC were enrolled from Tsinghua University Affiliated Beijing Tsinghua Chang Gung Hospital between January 2021 and August 2022. The inclusion criteria were as follows:(1) Confirmed diagnosis of HCC based on pathological findings, clinical features, and imaging studies. (2) Age above 18 years. (3) Estimated survival time exceeding 3 months. (4) No prior history of antitumor therapy. (5) Child‐Pugh classification of either A or B. (6) Eastern Cooperative Oncology Group (ECOG) performance status (PS) of 0 or 1. The exclusion criteria included: (1) Presence of severe medical comorbidities, such as cardiac or renal dysfunction. (2) Active infection. (3) Concurrent or historical diagnosis of malignancies other than HCC. Additionally, patients diagnosed with liver cirrhosis (LC group) and healthy controls (HC group) were recruited. The diagnosis of liver cirrhosis was established based on clinical and laboratory findings, in accordance with the 2019 Chinese guidelines on the management of liver cirrhosis issued by the Chinese Society of Hepatology.^12^

All participants provided written informed consent before their inclusion in the study. The study protocols were approved by the Ethics Committee of Tsinghua University Affiliated Beijing Tsinghua Chang Gung Hospital.

### 2.2 mTACE procedure

The mTACE procedure employed gelatin sponge microparticles (GSMs) of varying sizes (150−350 µm, 350−560 µm, and 560−710 µm, supplied by Hanzhou Alc Ltd.) as embolic agents. The choice of GSM size was determined based on the diameter of the tumor‐feeding arteries, tumor size, and degree of tumor staining, as described in previous studies.^10^, ^11^, ^13^, ^14^ The appropriate dosage of epirubicin was selected according to tumor size: 30 mg for tumors smaller than 5 cm and 50 mg for tumors larger than 5 cm. Epirubicin was diluted in 50‐100 mL of saline and mixed with the GSMs. The mixture was then slowly injected into the tumor‐feeding artery until complete disappearance of tumor staining was observed.

### 2.3 Clinical information

Demographic variables, including age and gender, were collected for all participants. Clinical parameters such as hepatitis B or C status, liver cirrhosis diagnosis, Barcelona Clinic Liver Cancer (BCLC) stage, liver function metrics, primary tumor diameter, alpha‐fetoprotein (AFP) levels, prothrombin induced by vitamin K absence‐II (PIVKA‐II), and the presence of portal vein invasion or distant metastasis were meticulously recorded before the mTACE procedure.

Post‐mTACE radiographic tumor responses were assessed 3−5 weeks following the procedure, employing the modified Response Evaluation Criteria in Solid Tumors (mRECIST) categories.^15^ The categories included Complete Response (CR), Partial Response (PR), Stable Disease (SD), and Progressive Disease (PD).

### 2.4 mMDSCs and eMDSCs evaluations

**Figure 1:**
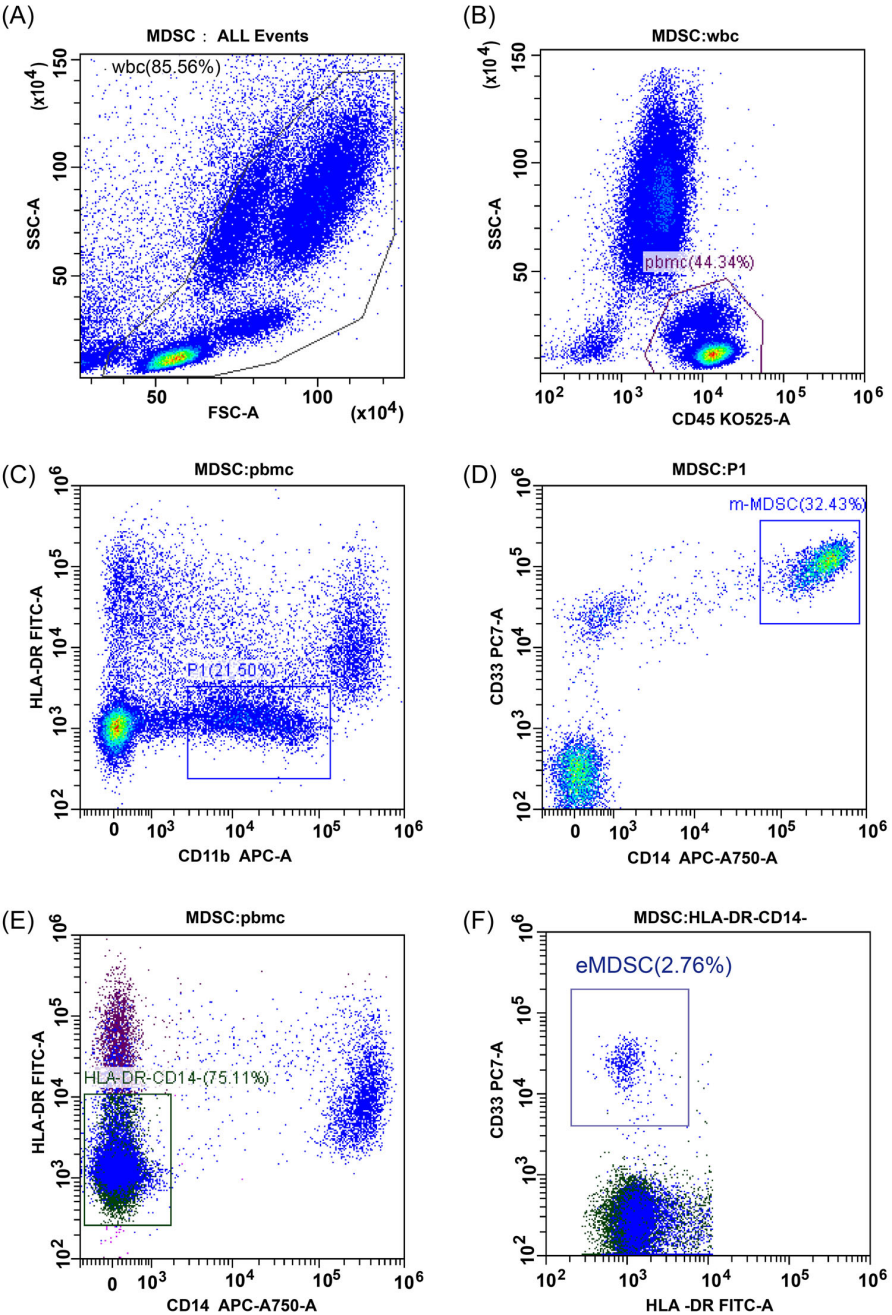
Gating Strategy for Identifying MDSC Subsets in Peripheral Blood. The final frequencies of mMDSC and eMDSC in this patient were 6.97% (21.50% × 32.43% × 100) and 2.07% (75.11% × 2.76% × 100), respectively. (A) Fresh whole blood (WB) samples served as the substrate for the flow cytometric assay. Boxes indicate the cell populations selected for further analysis, with an initial exclusion of cell debris. (B) Live PBMC cells were selected using plots of CD45 versus SSC‐A. (C, D) Gating strategy for mMDSCs. The P1 box was selected by gating on cells positive for CD11b and negative or low‐negative for HLA‐DR antibodies. Subsequently, mMDSCs were selected by gating on CD14+ cells and CD33+ cells. (E, F) Gating strategy for eMDSCs. Based on the PBMC box (Figure 1B), cells were selected by gating on cells negative for CD14 and HLA‐DR antibodies. Subsequently, eMDSCs were selected by gating on CD33+ cells using plots of CD33 versus HLA‐DR. eMDSCs, early‐stage myeloid‐derived suppressor cells; PBMCs, peripheral blood mononuclear cells.

Peripheral blood samples were obtained from all study participants. For the HCC patient cohort, samples were collected 1 day before mTACE and again 3−5 weeks postprocedure. In this study, eMDSCs were identified as HLA‐DR−/CD14−/CD33+ and mMDSCs as CD11b+/CD33+/CD14+/HLA‐DRlow/−.^3^, ^16^, ^17^ Multicolor fluorescence‐activated cell sorting analysis was conducted within 4 h of blood collection to determine the frequencies of mMDSCs and eMDSCs. A 100 μL aliquot of peripheral blood was stained with a panel of monoclonal antibodies, including CD11b‐APC‐Alexa Fluor 750, CD14‐PC7, CD33‐APC‐A750, and anti‐HLA‐DR‐ECD (Beckman Coulter, Miami, FL, USA). Cells were lysed using 500 mL of red blood cells lysing solution (Beckman Coulter) and incubated at 25°C for 15 min. Following two wash cycles, samples were analyzed using the Beckman Coulter DxFLEX Flow Cytometer version 1.1 system. We chose the preoperative test results of one patient with HCC to illustrate the selective pass strategy for mMDSCs and eMDSCs, as depicted in Figure 1. The results revealed that the frequencies of preoperative mMDSCs and eMDSCs in the patient were 6.97% and 2.07%, respectively. Consequently, the overall frequency of all tested MDSCs was expressed as a percentage of peripheral blood mononuclear cells.

### 2.5 Statistical analysis

The frequencies of mMDSCs and eMDSCs were reported as mean ± standard deviation (SD). For comparative analyses, both paired and unpaired t‐tests were employed to evaluate the differences in mMDSCs and eMDSCs frequencies between the HCC, LC, and HC groups, as well as among HCC patients with varying clinical characteristics. To investigate the associations between MDSCs frequencies and clinical parameters, ordinary least squares regression analyses were conducted. Specifically, the frequency of mMDSCs was regressed against the value of AFP, the value of PIVKA‐II, and the diameter of the tumor. Given the wide range of values for AFP and PIVKA‐II, logarithmic transformations were applied to these variables to achieve a more normalized distribution for regression analysis. All statistical analyses were executed using GraphPad Prism version 9.3.1 (GraphPad Software Inc.). A *p* Value threshold of <0.05 was established for statistical significance.

## 3 RESULTS

### 3.1 Patient profiles

A total of 75 HCC patients (HCC group), 16 liver cirrhosis patients (LC group), and 20 healthy controls (HC group) were enrolled in this study. Within the HCC cohort, 58 patients were diagnosed with chronic hepatitis B, including 15 with positive HBeAg and 43 with negative HBeAg. The remaining 17 HCC patients consisted of 4 with hepatitis C virus (HCV) and 13 without either HBV or HCV infection. Supporting Information S1: Table 1 provides a comprehensive summary of all clinical information, including tumor diameters and BCLC stages, for the patients in the mTACE group.

### 3.2 Frequency of mMDSCs and eMDSCs across groups

**Figure 2:**
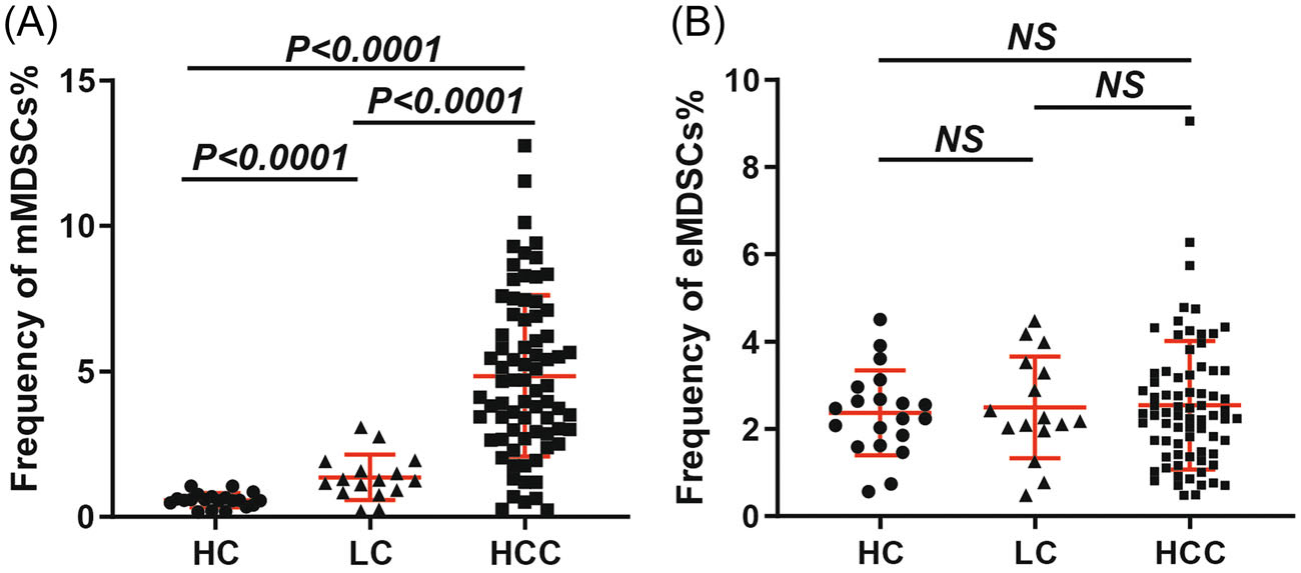
. Frequency of MDSCs in the Health Control (HC) Group, Liver Cirrhosis (LC) Group, and hepatocellular carcinoma (HCC) Group. A) Represents the frequency of mMDSCs. B) Represents the frequency of eMDSCs. eMDSCs, early‐stage myeloid‐derived suppressor cells; MDSCs, myeloid‐derived suppressor cells.

As delineated in Figure 2 and Supporting Information S2: Table 2, the frequency of mMDSCs in the HCC group (4.85 ± 2.77%) was significantly elevated compared to the LC group (1.36 ± 0.78%) and HC group (0.57 ± 0.25%). Conversely, no statistically significant differences were observed in the frequency of eMDSCs among the three groups.

### 3.3 Associations between MDSCs subtypes and clinical characteristics in HCC patients

**Figure 3:**
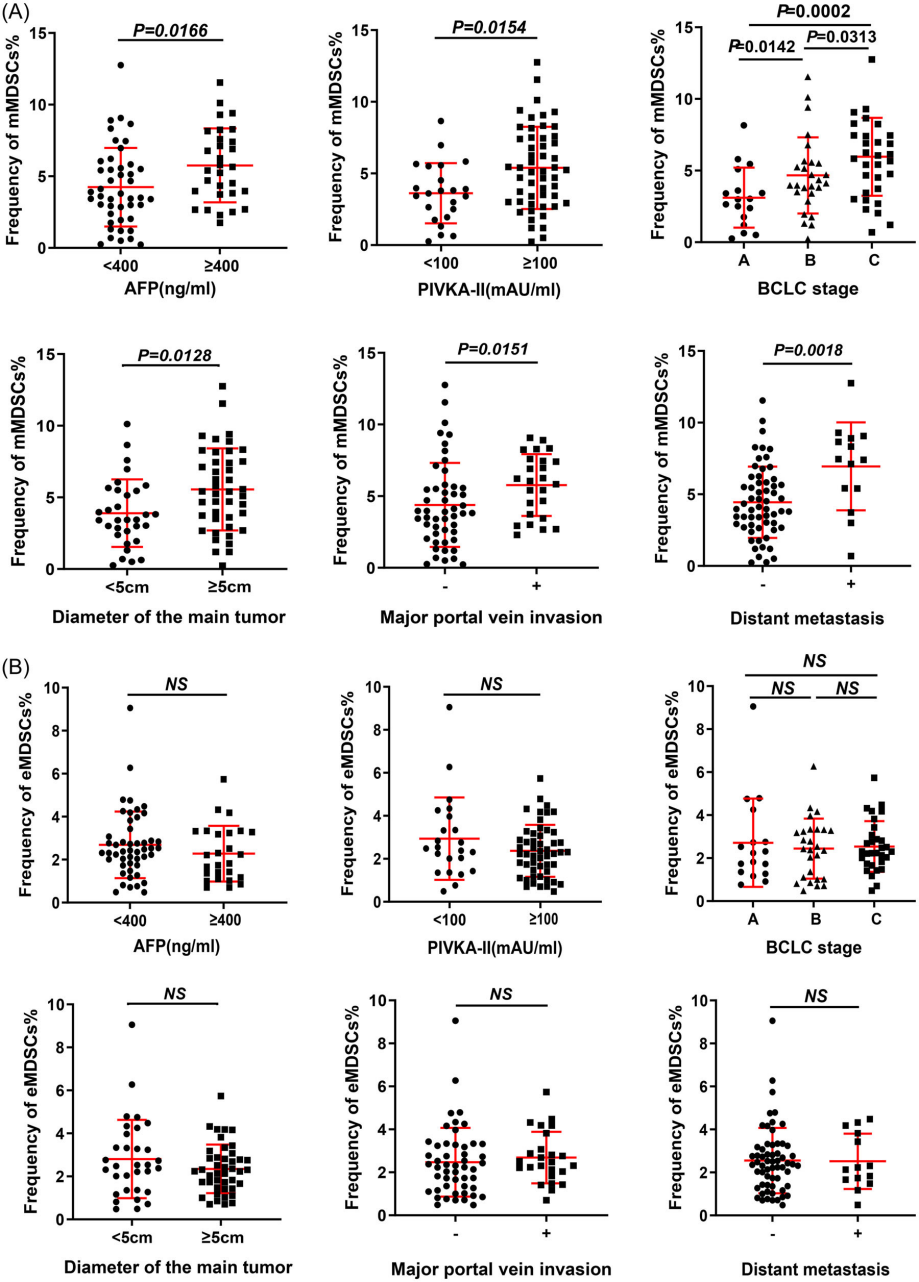
. The comparison of MDSCs frequencies between different tumor characteristics. (A) The frequency of mMDSCs in HCC patients with different clinical characteristics; (B) The frequency of eMDSCs in HCC patients with different clinical characteristics. eMDSCs, early‐stage myeloid‐derived suppressor cells; HCC, hepatocellular carcinoma; MDSCs, myeloid‐derived suppressor cells.

Figure 3 and Supplementary Table 3 present the frequencies of mMDSCs and eMDSCs in HCC patients stratified by various clinical parameters. As elegantly demonstrated in Figure 3A, the frequency of mMDSCs distinctly surged in HCC patients with an AFP level exceeding 400 ng/mL, in stark contrast to those with AFP levels at or below 400 ng/mL (5.76 ± 2.58% vs. 4.24 ± 2.74%, *p* = .0166). This intriguing trend persisted when comparing patients with PIVKA‐II levels surpassing 100 mAU/ml to those below this threshold (5.39 ± 2.78% vs. 3.62 ± 2.10%, *p* = .0154). Furthermore, a compelling correlation materialized between the frequency of mMDSCs and the stage of HCC. Patients grappling with BCLC stage C exhibited a strikingly elevated mMDSCs level (5.96 ± 2.71%) compared to those at stages A (3.11 ± 2.10%) and B (4.67 ± 2.66%) (*p* = .0002 and *p* = .0313, respectively). Additionally, mMDSCs prevalence soared in HCC patients with a primary tumor size exceeding 5 cm when contrasted with those harboring tumors of 5 cm or less (5.56 ± 2.86% vs. 3.90 ± 2.35%, *p* = .0128). A similar pattern emerged when major portal vein invasions were considered, as patients with vascular invasions displayed a substantially higher mMDSCs frequency (6.95 ± 3.07% vs. 4.44 ± 2.48%, *p* = .0151). Correspondingly, HCC patients grappling with tumor metastasis exhibited a pronouncedly heightened frequency of mMDSCs in comparison to their non‐metastatic counterparts (6.04 ± 3.42% vs. 4.58 ± 2.69%, *p* = .0018). It is worth noting that for eMDSCs, no statistically significant distinctions were observed among the various subgroups within the HCC patient cohort (Figure 3B).

**Figure 4:**
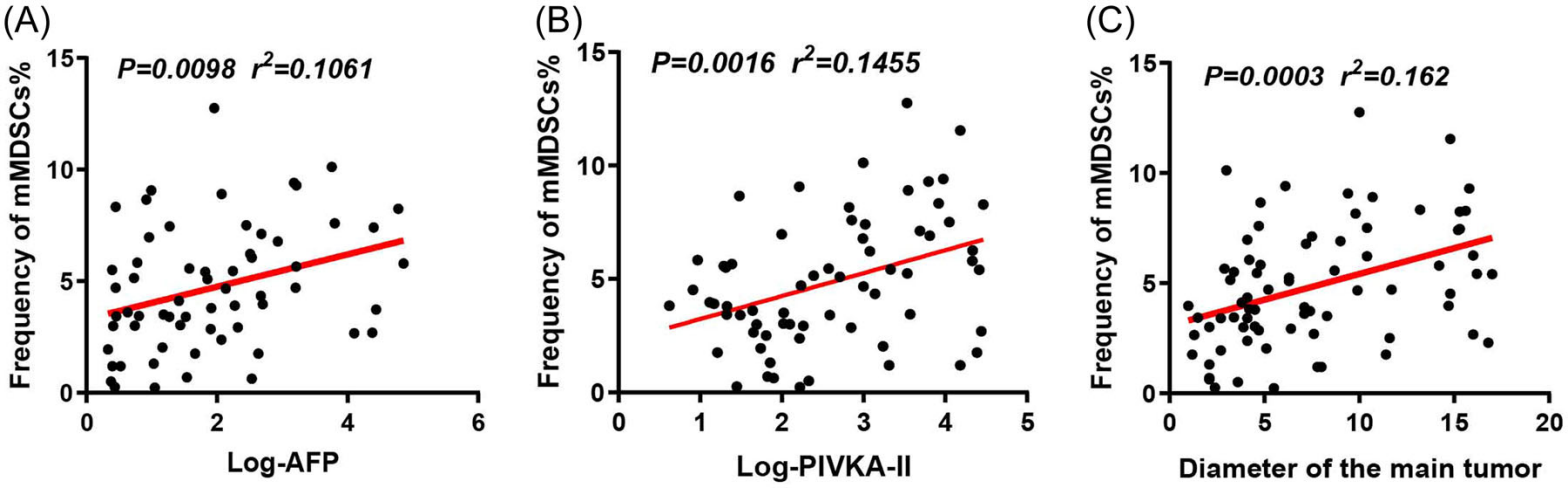
. Associations between mMDSCs Abundance and Clinical Parameters in HCC Patients. (A) Depicts the association between the abundance of mMDSCs and the serum level of AFP in HCC patients. The equation describing this relationship is: mMDSCs (%) = 0.7241 * log(AFP) (ng/mL) + 3.306. (B) Illustrates the association between the abundance of mMDSCs and the serum level of PIVKA‐II in HCC patients. The equation for this relationship is: mMDSCs (%) = 1.011 * log(PIVKA‐II) (mAU/ml) + 2.224. (C) Demonstrates the association between the abundance of mMDSCs and the major tumor size in HCC patients. The equation representing this correlation is: mMDSCs (%) = 0.2338 * diameter (cm) + 3.076. AFP, alpha‐fetoprotein; HCC, hepatocellular carcinoma; MDSCs, myeloid‐derived suppressor cells; PIVKA‐II, prothrombin induced by vitamin K absence‐II.

Notably, elevated frequencies of mMDSCs were associated with higher AFP levels, elevated PIVKA‐II values, advanced BCLC stages, larger tumor sizes, and the presence of portal vein invasion or distant metastasis. However, no such associations were observed for eMDSCs. Regression analyses revealed positive correlations between serum levels of AFP and PIVKA‐II and the frequency of mMDSCs, as illustrated in Figures 4A,B. Additionally, a positive correlation was observed between tumor diameter and mMDSCs frequency (Figure 4C).

### 3.4 The impact of mTACE on mMDSCs, eMDSCs, AFP levels, and patient survival

**Figure 5:**
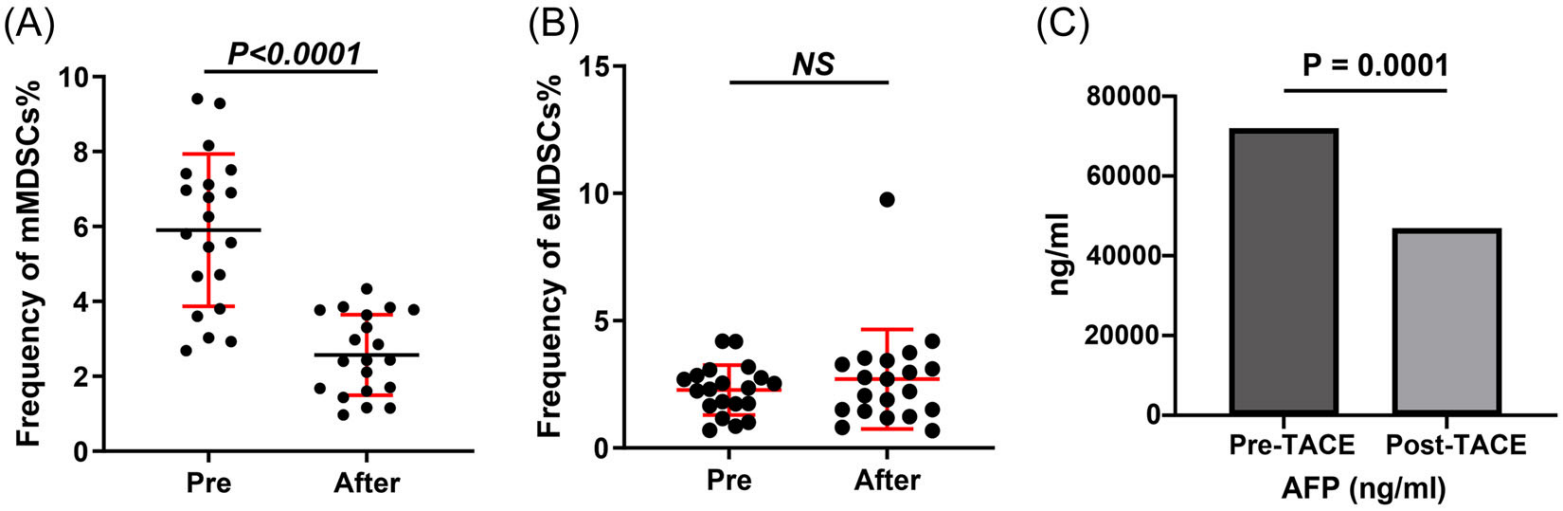
Alterations in Peripheral Blood MDSCs Frequencies Among HCC Patients in Response to mTACE Treatment. (A) Depicts the quantification of changes in mMDSCs (Monocytic MDSCs) frequencies before and after mTACE. (B) Depicts the quantification of changes in eMDSCs (Granulocytic MDSCs) frequencies before and after mTACE. (C) Changes in AFP Levels before and after mTACE. eMDSCs, early‐stage myeloid‐derived suppressor cells; MDSCs, myeloid‐derived suppressor cells; mTACE, Microparticle Transarterial Chemoembolization.

As illustrated in Figure 5 and Supporting Information S1: Table 4, a marked reduction in the frequency of mMDSCs was observed following mTACE treatment, decreasing from 5.90 ± 2.03% to 2.57 ± 1.07% (*p* < .0001). Conversely, the frequency of eMDSCs remained statistically unchanged post‐mTACE (2.28 ± 0.98% vs. 2.70 ± 1.96%, *p* = .4474). We also compared the changes in AFP levels before and after TACE in these 20 patients. The results showed a significant difference in AFP levels after TACE (*p* = .0001), as illustrated in Figure 5C.

**Figure 6:**
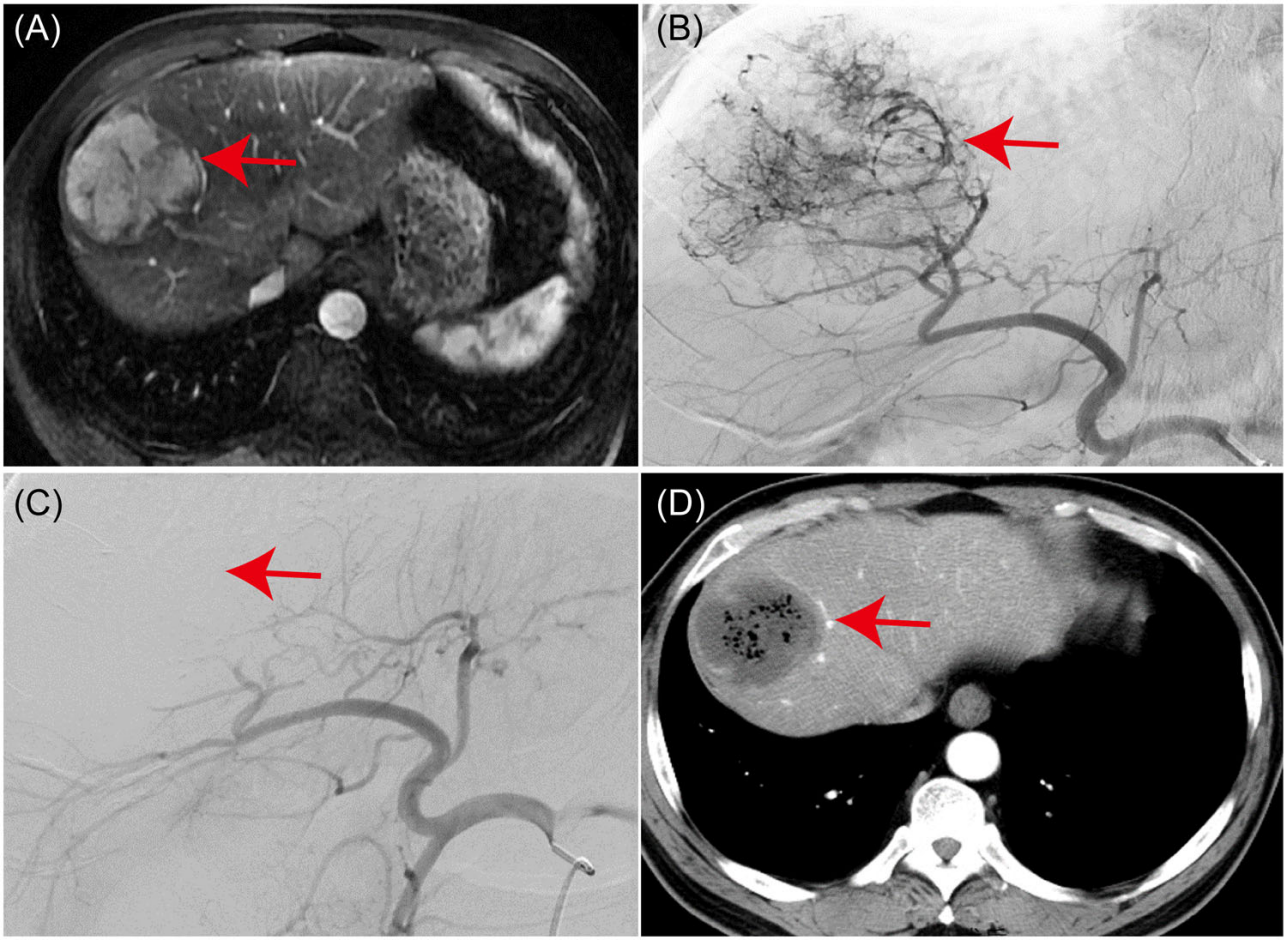
Response to mTACE Treatment in a 62‐Year‐Old Male Patient with a Massive HCC. The area indicated by the red arrow is where the tumor is located. (A) Enhanced MRI of the upper abdomen displaying a substantial mass with evident enhancement during the arterial phase, situated in the right lobe of the liver. The maximum diameter of the mass measures 4.1 cm. (B) Angiography depicting extensive tumor staining in the right lobe of the liver before mTACE intervention. (C) Post‐mTACE, the tumor's blood supply artery exhibits near‐complete occlusion. (D) Enhanced CT scan of the upper abdomen conducted 4 weeks following mTACE, illustrating significant tumor liquefaction and necrosis. eMDSCs, early‐stage myeloid‐derived suppressor cells; HCC, hepatocellular carcinoma; MDSCs, myeloid‐derived suppressor cells; mTACE, Microparticle Transarterial Chemoembolization.

All patients were discharged postoperatively and underwent regular follow‐up at outpatient clinics. All patients survived until the last follow‐up. Among the 20 patients with complete diagnostic imaging, four achieved a complete response (CR, 20%), while 16 achieved a partial response (PR, 80%), yielding an overall response rate of 100%. The mean follow‐up duration was 13.6 ± 6.0 months, with the longest being 25 months. The patient depicted in Figure 6 had an initial mMDSCs frequency of 6.97% and an eMDSCs frequency of 2.07%. Following mTACE, a significant reduction in mMDSCs to 3.78% was observed, while eMDSCs levels remained stable at 2.55%.

Ten out of 20 patients who underwent subsequent immunotherapy post‐mTACE achieved a 1‐year PFS rate of 90% (Nine patients who received immunotherapy did not show progression during regular 1‐year follow‐up. Progression of intrahepatic disease was detected in another patient at the ninth month of follow‐up). In contrast, the 1‐year PFS rate was 60% among those who did not receive immunotherapy(Intrahepatic disease progression was detected at the 1‐year follow‐up period in four of the patients who did not undergo immunotherapy after TACE treatment, respectively). Preliminary data suggest that the combination of mTACE and immunotherapy may offer a survival advantage, particularly for patients with advanced HCC stages, as evidenced by the higher number of BCLC stage C patients in the mTACE plus immunotherapy group (Supporting Information S1: Figure 1).

## 4 DISCUSSION

MDSCs have garnered increasing attention in HCC research over the past decade. Our study corroborates previous findings that the frequency of CD11b+/CD33+/CD14+/HLA‐DRlow/− mMDSCs is significantly elevated in HCC patients compared to cirrhotic patients and HC.^6^, ^18^, ^19^ Interestingly, we observed an elevated frequency of mMDSCs in cirrhotic patients compared to HC, diverging from Arihara F's study.^20^ This discrepancy warrants further investigation. Our data, in conjunction with our prior work on the decreased frequency of regulatory T cells (Tregs) and altered CD4/CD8 ratios,^21^ underscore the compromised immunological landscape in HCC, necessitating immunomodulatory strategies during treatment.

Our findings align with existing literature in suggesting that elevated mMDSCs levels are indicative of aggressive HCC phenotypes, including advanced BCLC stages, elevated serum AFP and PIVKA‐II levels, and the presence of portal vein tumor thrombus and metastases.^2^, ^6^, ^22^, ^23^, ^24^ Given the ease of peripheral blood sampling, MDSCs could serve as a complementary biomarker to AFP for assessing tumor biology and guiding therapeutic strategies. eMDSCs, a less characterized and immature subpopulation of MDSCs, did not show any significant association with HCC biology in our study. This highlights the need for further research to elucidate the role of eMDSCs in HCC immunosuppression.

Our study is among the first to report changes in MDSC frequencies following mTACE treatment, revealing a significant reduction in mMDSCs post‐treatment.^25^ This suggests that mTACE‐induced tumor necrosis and burden reduction may contribute to this decrease, although this is not universally observed across all treatment modalities for HCC.^20^, ^24^, ^26^, ^27^ The differential impact of mTACE and other treatments like RFA on MDSC frequencies merits further investigation.

Recent studies have demonstrated that lower mMDSCs levels are associated with better outcomes in patients with advanced NSCLC receiving anti‐PD‐1 immunotherapy.^28^ Our preliminary data suggest that mTACE, by reducing mMDSCs levels, may create a more favorable environment for subsequent immunotherapies, such as anti‐PD‐1 inhibitors and CAR‐T therapies. This is supported by our observation that patients receiving post‐mTACE immunotherapy had a 1‐year PFS rate of 90%, compared to 60% in those who did not.

Our study has several limitations, including a small sample size and the absence of data on overall survival. Additionally, we did not explore the tumor microenvironment, focusing only on peripheral blood. Future studies should aim to expand the sample size, investigate the tumor microenvironment, and assess the long‐term efficacy of combination therapies.

## 5 CONCLUSIONS

In this comprehensive study, we have uncovered a novel role for mMDSCs as potential complementary biomarkers alongside AFP. Through an in‐depth analysis of these cells, we made a significant discovery: the frequency of mMDSCs exhibited a substantial reduction following mTACE. This observation holds promising implications for the future of immunotherapy in HCC management interventions.

## AUTHOR CONTRIBUTIONS

*Conceptualization*: Yuanxun Yue and Yuewei Zhang. *Data curation*: Zhizhong Ren and Tianxiao Wang. *Formal analysis*: Yuanxun Yue, Zhizhong Ren, Yaqin Wang, Sujun Li, Qian Chen, Yating Bai, and He Zhou. *Investigation*: Ying Liu and Xiaowei Yang. *Methodology*: Yuanxun Yue, Zhizhong Ren, and Yaqin Wang. *Project administration*: Yuewei Zhang and Ying Liu. *Software*: Yuanxun Yue and Zhizhong Ren. *Supervision*: Yaqin Wang, Ying Liu, Xiaowei Yang, and Yuewei Zhang. *Validation*: Yuewei Zhang. *Writing original draft*: Yuanxun Yue. *Writing review and editing*: Zhizhong Ren, Yaqin Wang, and Yuewei Zhang.

## CONFLICT OF INTEREST STATEMENT

The authors declare no conflict of interest.

## Supporting information

Supporting information.

Supporting information.

## Data Availability

The data that support the findings of this study are available from the corresponding author upon reasonable request.
