# Supplementary figures and images for: Impact of Microparticle Transarterial Chemoembolization (mTACE) on myeloid‐derived suppressor cell subtypes in hepatocellular carcinoma: Clinical correlations and therapeutic implications
Source: Immun Inflamm Dis. 2024 Sep 2;12(9):e70007. doi: 10.1002/iid3.70007 (PMC11367920; doi:10.1002/iid3.70007)

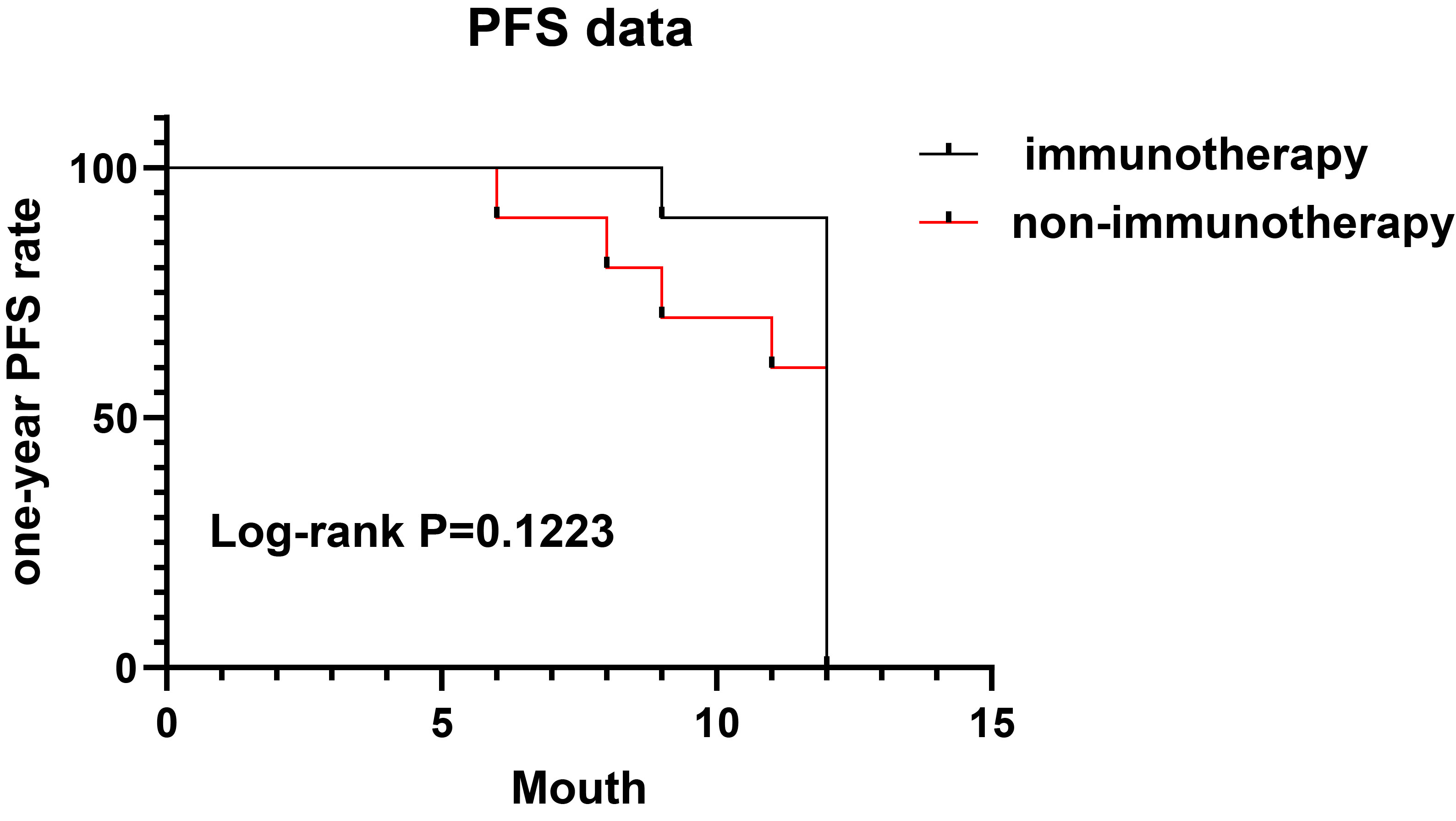

Supplement: Supplementary file 1 — Supporting information. [file IID3-12-e70007-s001.jpg]
