# Supplementary material for: Impact of Microparticle Transarterial Chemoembolization (mTACE) on myeloid‐derived suppressor cell subtypes in hepatocellular carcinoma: Clinical correlations and therapeutic implications
Source: Immun Inflamm Dis. 2024 Sep 2;12(9):e70007. doi: 10.1002/iid3.70007 (PMC11367920; doi:10.1002/iid3.70007)
Supplement: Supplementary file 2 — Supporting information. [file IID3-12-e70007-s002.docx]

**
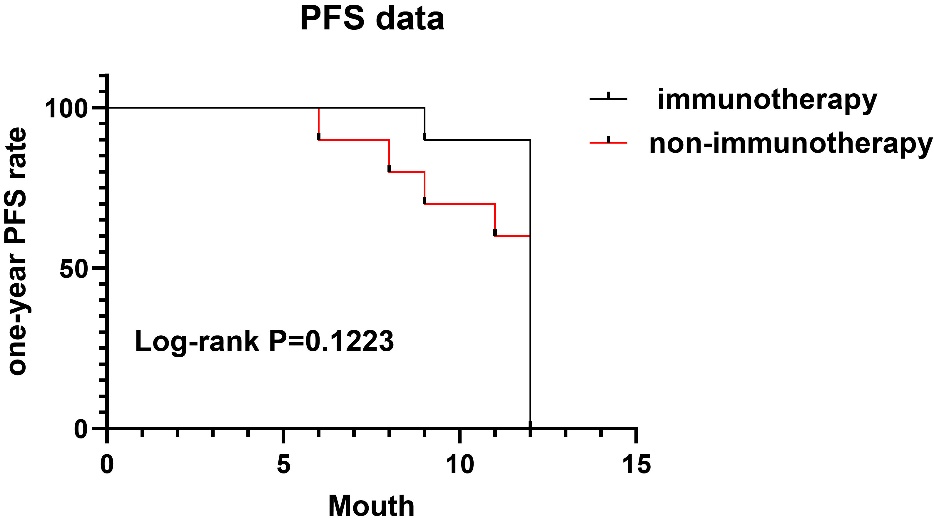
**

**Supplementary figure 1.** The comparison of survival periods between patients who received immunotherapy and those who did not.

**Supplementary 1.** Characteristics of patients with HCC and individuals included in mTACE analysis.

| ***Parameters*** | ***HCC (N=75)*** | ***mTACE (N=20)*** |
| --- | --- | --- |
| **Age, years (range)** | 57 (13-86) | 58(37-78) |
| **Gender, N** |  |  |
| Male | 64 | 17 |
| Female | 11 | 3 |
| **chronic hepatitis B, N** |  |  |
| CHB | 58 | 16 |
| Non-CHB | 17 | 4 |
| **HCV, N** |  |  |
| Positive | 4 | 1 |
| Negative | 71 | 19 |
| **Main tumor diameter, mm (range)** | 75.81 (10-170) | 90.15(41-160) |
| **BCLC staging (A/B/C), N** | 17/27/31 | 5/6/9 |
| **Liver function (Child A/B/C), N** | 67/8/0 | 19/1/0 |
| **Distant metastasis, N** |  |  |
| Positive | 14 | 3 |
| Negative | 61 | 17 |
| **Major portal vein invasion, N** |  |  |
| Positive | 25 | 7 |
| Negative | 50 | 13 |

**Supplementary 2.** Comparisons of the abundances of MDSCs among the HC group, LC group, and HCC group.

| **Group** | ***N*** | ***mMDSCs*** | ***p*** | ***eMDSCs*** | ***p*** |
| --- | --- | --- | --- | --- | --- |
| **HC** | 20 | 0.57 ± 0.25% | - | 2.37 ± 0.98% | - |
| **LC** | 16 | 1.36 ± 0.78 % | <0.0001*(vs. HC)* | 2.49 ± 1.17% | ＞0.05*(vs. HC)* |
| **HCC** | 75 | 4.85 ± 2.77% | <0.0001*(vs. LC)* | 2.54 ± 1.47% | ＞0.05 *(vs. LC)* |

**Supplementary 3.** The association between the MDSC proportion with clinical characteristics of HCC patients.

| ***Characteristics*** | ***N*** | ***mMDSCs (%)*** | ***p*** | ***eMDSCs (%)*** | | ***p*** |
| --- | --- | --- | --- | --- | --- | --- |
| **AFP** |  |  |  |  | |  |
| ≤400 ng/mL | 49 | 4.24 ±2.74 | 0.0166 | 2.69 ± 1.55 | | 0.2408 |
| >400 ng/mL | 26 | 5.76 ± 2.58 |  | 2.28± 1.30 | |  |
| **PIVKA-II** |  |  |  |  | |  |
| ≤100mAU/ml | 23 | 3.62 ± 2.10 | 0.0154 | 2.94 ± 1.92 | | 0.2888 |
| >100mAU/ml | 52 | 5.39 ± 2.78 |  | 2.37 ± 1.21 | |  |
| **BCLC staging** |  |  |  |  |  | |
| A | 17 | 3.11 ± 2.10 |  | 2.71 ± 2.05 | |  |
| B | 27 | 4.67 ± 2.66 | 0.0142 (vs. A) | 2.44 ± 1.39 | | 0.9953(vs. A) |
| C | 31 | 5.96 ± 2.71 | 0.0002 (vs. A) | 2.54 ± 1.18 | | 0.6734 (vs. A) |
| **Main tumor diameter** |  |  |  |  | |  |
| ≤5 cm | 32 | 3.90 ± 2.35 | 0.0128 | 2.81 ± 1.52 | | 0.3549 |
| >5 cm | 43 | 5.56 ± 2.86 |  | 2.35 ± 1.13 | |  |
| **Major portal vein invasion** |  |  |  |  | |  |
| No | 50 | 4.44 ± 2.48 | 0.0151 | 2.47 ± 1.60 | | 0.3100 |
| Yes | 25 | 6.95 ± 3.07 |  | 2.69 ± 1.20 | |  |
| **Tumor metastasis** |  |  |  |  | |  |
| No | 61 | 4.58 ± 2.69 | 0.0018 | 2.55 ± 1.52 | | 0.9973 |
| Yes | 14 | 6.04 ± 3.42 |  | 2.52 ± 1.29 | |  |

**Supplementary 4.** Clinical characteristics and MDSCs data of HCC patients in pre- and post-mTACE analysis.

| **Case#** | ***Diameter of the main tumor (mm)*** | ***BCLC staging (A/B/C)*** | ***Major portal vein invasion (+/-)*** | ***Distant metastasis***  ***(+/-)*** | ***AFP (ng/ml)***  ***(Pre-TACE)*** | ***mRECIST*** | ***mMDSCs (%)*** | | ***eMDSCs (%)*** | |
| --- | --- | --- | --- | --- | --- | --- | --- | --- | --- | --- |
|  |  |  |  |  |  |  | ***Pre*** | ***Post*** | ***Pre*** | ***Post*** |
| 1 | 71 | A | - | - | 4.3 | CR | 3.60 | 1.60 | 1.82 | 0.75 |
| 2 | 45 | A | - | - | 27.32 | PR | 3.03 | 1.68 | 0.86 | 0.48 |
| 3 | 142 | A | - | - | 71907.93 | PR | 5.80 | 2.85 | 1.31 | 1.78 |
| 4 | 104 | B | - | - | 280.47 | CR | 7.51 | 2.98 | 1.89 | 0.83 |
| 5 | 45 | B | - | - | 81.55 | PR | 3.80 | 1.70 | 1.69 | 1.80 |
| 6 | 61 | B | - | - | 1505.14 | PR | 9.41 | 2.40 | 2.02 | 2.29 |
| 7 | 117 | B | - | - | 1602.29 | PR | 4.71 | 3.64 | 0.55 | 1.75 |
| 8 | 76 | C | + | - | 24005.9 | PR | 2.69 | 1.44 | 0.62 | 1.26 |
| 9 | 64 | C | + | - | 208.37 | PR | 2.93 | 1.15 | 1.78 | 1.34 |
| 10 | 41 | C | + | - | 9.05 | PR | 6.97 | 3.78 | 2.07 | 2.55 |
| 11 | 152 | C | + | + | 25061 | PR | 7.41 | 3.77 | 2.31 | 6.91 |
| 12 | 160 | C | + | - | 2.00 | PR | 6.26 | 0.97 | 1.92 | 2.04 |
| 13 | 158 | C | - | + | 1647.46 | PR | 9.29 | 2.11 | 1.28 | 1.86 |
| 14 | 99 | C | + | - | 136.04 | PR | 4.67 | 1.16 | 2.50 | 2.02 |
| 15 | 46 | A | - | - | 175.05 | CR | 5.45 | 2.44 | 1.63 | 2.13 |
| 16 | 87 | B | - | - | 37.71 | PR | 5.57 | 2.43 | 2.84 | 1.90 |
| 17 | 98 | A | - | - | 2.00 | PR | 8.16 | 3.84 | 1.73 | 1.45 |
| 18 | 72 | B | - | - | 854.87 | CR | 6.78 | 3.85 | 1.00 | 1.18 |
| 19 | 90 | C | + | - | 502.75 | PR | 6.90 | 3.30 | 2.24 | 1.23 |
| 20 | 75 | C | - | + | 479.25 | PR | 7.12 | 4.34 | 1.66 | 0.68 |
